# The Advantages to Be Gained by the Administration of Morphia in Certain Conditions

**Published:** 1907-10-26

**Authors:** B. J. Courtney


### The General Practitioner's Column.

[Contributions to this Column are invited, and if accepted will be paid for.]

### MORPHIA IN CERTAIN CONDITIONS.

By B. J. COURTNEY, M.D., B.Ch.

There can be little doubt that in former years many practitioners made freer use of opium and its derivatives in the treatment of various diseases than the more scientific views of the present days would admit to have been advantageous. At the same time, one is sometimes led to think that the pendulum has now swung too far in the opposite direction, and that opium is now frequently withheld from a patient when it might be given with considerable advantage either in treatment or as an aid to diagnosis.

The chief reason for this decline in favour of a drug so well known, and with the properties of which we are so familiar, would appear to be the teaching of the operative surgeon, whose frequent reiteration of the statement regarding opium, that it masks the symptoms of acute abdominal diseases, has so impressed itself upon some minds that its administration under almost any circumstances is looked upon as little less than a crime. While not disputing for a moment that the administration of opium in such a disease as appendicitis is capable of obscuring symptoms, and thus of leading to a loss of valuable time, yet at the same time it should be borne in mind —and this is what is so frequently forgotten—that the giving of opium in certain diseases not only does not obscure the diagnosis, but may be a distinct and direct aid in diagnosis. Consequently the cases in which opium appears to be indicated, and from which it is proposed to be withheld on the grounds of masking the symptoms, should be very carefully selected, each being considered on its individual merits, and not on general principles. A few examples of wrong diagnoses through withholding opium in cases of acute disease in the cavity of the abdomen or pelvis may serve to illustrate the need for exercising great care before electing to pursue such a line of treatment.

Case 1.—A woman, aged 53, was seized suddenly during the night with acute pains in the abdomen, chiefly on the right side and in the iliac region; and with vomiting, which was repeated at intervals for several hours; any solid or liquid taken was immediately rejected. On examination the abdomen was found to be exceedingly tender, and the muscles became rigid on the slightest attempt at palpation, the results of which were consequently negative; there was nothing abnormal to be seen on inspection: a provisional diagnosis of appendicitis was made, and the patient was advised that she ought to be removed to hospital with a view to operation. Before taking this advice the patient sought a second opinion, with the result that a suppository of ¼ gr. of morphia was given per rectum; the patient was much relieved, the vomiting ceased, and after a few hours a complete examination of the abdomen and pelvis was possible, with the result that a small ovarian cyst on the right side was discovered, and it was surmised that the acute symptoms above noted were due to twisting of the pedicle and local peritonitis—a diagnosis which was confirmed at the operation, which was done a week later. In this case, therefore, the correct diagnosis was arrived at only after the pain was relieved by morphia: that is to say, that the morphia not only did not obscure the diagnosis, but very materially aided it, besides greatly relieving the urgent symptoms of the patient.

Case 2.—A lady, aged 40, developed a septic infection of the uterus, with the usual symptoms, following a miscarriage. Soon after these symptoms had subsided she suffered severe pain in the left side of the pelvis, with a rise of temperature to 103°-104° in the evening, with morning remissions. These symptoms, with occasional vomiting, led to the suspicion of abscess formation in the pelvis. In addition the constant pain and loss of rest gave rise to great exhaustion, and it was not until a hypodermic of morphia was given, and the patient thereby quieted that a proper examination could be made, and a diagnosis of simple pelvic cellulitis arrived at, the symptoms of which were aggravated by the neurotic and exhausted condition of the patient. This opinion the subsequent course of the case upheld.

Case 3.—A child, aged 5, developed a sharp attack of acute lobar pneumonia. On the second day of the illness vomiting of a severe and intractable nature set in, accompanied by insomnia. All the usual remedies for both these symptoms were tried, and proved entirely useless, and it seemed probable that they would bring about a fatal termination. Two minims of liq. morph. hydrochlor. were ordered every two hours in water; after the third or fourth dose the vomiting ceased, and the child had several hours sleep. The subsequent course of the case was uneventful and satisfactory.

These few examples may serve to show that morphia administered in suitable cases may be of inestimable value in both treatment and diagnosis, and—while fully admitting the teaching that valuable time may be lost by masking the symptoms of some acute abdominal conditions with morphia—also to remind us that this rule applies only to some diseases and not to all. Provided these do not exist it is neither the proper nor the best treatment to allow a patient to remain in unnecessary pain; and, further, the administration of morphia is in many cases necessary either as an aid in diagnosis or as a means of treatment.

When one has frequently seen cases of biliary colic, of pelvic peritonitis, of intestinal colic, to mention a few instances, and even of inoperable cancer, left in unnecessary pain through a wrongful appreciation of the teaching about masking the symptoms with morphia, one cannot help thinking that the other side of the question might, with advantage, be given more consideration.

